# Supplementary material for: Human RNase H2 upregulation counteracts oncogene- and chemotherapy-induced replication stress
Source: Oncogene. 2025 Jul 10;44(35):3255–71. doi: 10.1038/s41388-025-03489-8 (PMC12375501; doi:10.1038/s41388-025-03489-8)
Supplement: Supplementary file 1 — Supplemental Figures and Tables [file 41388_2025_3489_MOESM1_ESM.docx]

**Human RNase H2 subunit upregulation counteracts oncogene- and chemotherapy-induced replication stress**

Rosanna J. Wilkins, Abirami Kannan, Siobhan A. Plass, Claire Wilson, Richard D. W. Kelly, Claire H.M. Tang, Panagiotis Kotsantis, Martin A. M. Reijns, Aditi Kanhere, Eva Petermann

**Supplemental Figures**

**Figure S1. Validation of RNase H2 subunit antibodies and increased RNase H2 levels in response to replication stress.**

**(A)** Protein levels of RNASEH2B (RH2B), RNASEH2C (RH2C), and vinculin (loading control) in HCT116 or NCIH747 cells after 24 or 48 h transfection with non-targeting control siRNA (siNTC) or RH2B siRNA (siRH2B), or no transfection (no siRNA). **(B)** Protein levels of RH2B and RH2C and vinculin (loading control) in NCIH747 cells after 24 or 48 h transfection with non-targeting control siRNA (siNTC) or RH2C siRNA (siRH2C). **(C)** Protein levels of RNASEH2A (RH2A), RNASEH2B (RH2B), RNASEH2C, and Tubulin (loading control) in HCT116 cells 72 h after transfection with non-targeting control siRNA (siNTC), siRNA against RH2B (siRH2B) or R2HA (siRH2A). **(D)** Quantification of cell cycle distribution in BJ-HRAS^G12V^ cells with and without 24 or 72 h HRAS^G12V^ induction, based on measuring DNA content using propidium iodide staining and flow cytometry. N=3 (24h), N=4 (72h), N=6 (control). **(E)** Flow cytometry gating strategy for quantification of cell cycle distribution as in D. Cells were stained with propidium iodide. **(F)** Relative protein levels of RNASEH2A (RH2A), RH2B and RH2C after 72 h Cyclin E induction in U2OS cells, normalised to tetracycline on (con) for each protein. N=3. **(G)** Relative protein levels of RH2A, RH2B and RH2C in BJ-hTERT cells after 4 h treatment with 200 μM hydroxyurea (HU), 25 nM gemcitabine (GEM), 10 μM camptothecin (CPT), or DMSO. N = 3 (RH2A), N = 4 (RH2B), N = 2 (RH2C).

The means and SEM (bars) of independent experiments are shown. Asterisks indicate p-values (ANOVA, ns: not significant, ** p < 0.01, **** p < 0.0001).

**Figure S2. Representative Western blots of RNase H2 subunit levels after chemotherapy treatment in colon cancer cell lines.**

**(A)** Protein levels of RH2B, RH2C and tubulin (loading control) in HT55 cells after 2 h treatment with 200 μM hydroxyurea (HU) or 10 μM camptothecin (CPT). **(B)** Protein levels of RH2B, RH2C and vinculin (loading control) in HT55 cells after 2 h treatment with 25 nM gemcitabine (GEM). **(C)** Protein levels of RH2B, RH2C and vinculin (loading control) in NCIH747 cells after 2 h drug treatment with 200 μM HU or 10 μM CPT. **(D)** Protein levels of RH2B, RH2C and vinculin (loading control) in NCIH747 cells after 2 h treatment with 25 nM GEM or 10 μM CPT. **(E)** Protein levels of RH2B, RH2C and vinculin (loading control) in LS174T cells after 2 h treatment with 200 μM HU or 10 μM CPT. **(F)** Protein levels of RH2B, RH2C and vinculin (loading control) in LS174T cells after 2 h treatment with 25 nM GEM. **(G)** Protein levels of RH2B, RH2C and tubulin (loading control) in HCT116 cells after 2 h treatment with 200 μM HU or 10 μM CPT. **(H)** Protein levels of RH2B, RH2C and tubulin (loading control) in HCT116 cells after 2 h treatment with HU, CPT or GEM as indicated.

**Figure S3. RNASEH2B overexpression and RNase H2 activity.**

**(A)** Relative protein levels of RH2A, RH2B and RH2C in HCT116-RH2B cells after 48 h RH2B induction with doxycycline, normalised to control for each protein. Relative levels of endogenous and ectopic RH2B are indicated. Endogenous and ectopic RH2B were combined for statistical tests. N=7 (N=6 for RH2A). **(B)** Relative protein levels of RH2A, RH2B and RH2C in BJ-RH2B cells after 48 h RH2B induction with doxycycline, normalised to control for each protein. Relative levels of endogenous and ectopic RH2B are indicated. Endogenous and ectopic RH2B were combined for statistical tests. N=7. **(C)** RT-qPCR analysis of *RNASEH2A (RH2A), RNASEH2B (RH2B)*, and *RNASEH2C (RH2C)* expression in BJ-hTERT-RH2B cells after 48 h RH2B induction compared to no dox control. N=4. **(D)** DRD:DNA substrate converted over time during incubation with buffer only, whole cell extract from HCT116-RH2B cells with (RH2B 48h) or without (control) 48 h RH2B induction. N=4 **(E)** RNA:DNA substrate converted over time during incubation with buffer, whole cell extract from HCT116-RH2B cells with or without 48 h RH2B induction. N=2. **(F)** DRD:DNA substrate converted over time during incubation with buffer only, whole cell extract from BJ-RH2B cells with or without 48 h RH2B induction. N=5 **(G)** RNA:DNA substrate converted over time during incubation with buffer, whole cell extract from BJ-RH2B cells with or without 48 h RH2B induction. N=3.

The means and SEM (bars) of independent experiments are shown. Asterisks indicate p-values (ANOVA, ns: not significant, * p < 0.05, *** p < 0.001).

**Figure S4. Impact of RNASEH2B overexpression on RNA:DNA hybrid levels.**

**(A)** Quantification of slot blot analysis of RNA:DNA hybrid levels in genomic DNA from HCT116-RH2B cells after 50 min treatment with 200 μM hydroxyurea (HU), 10 μM CPT or DMSO as in Fig. 3F, with normalisation to DMSO for each condition (control or RH2B). N=4. **(B)** Quantification of slot blot analysis of RNA:DNA hybrid levels in genomic DNA from BJ-RH2B cells after 50 min treatment with 200 μM hydroxyurea (HU), 10 μM CPT or DMSO as in Fig. 3F, with normalisation to DMSO for each condition (control or RH2B). N=6. **(C)** Protein levels of RH2A, RH2B, RH2C, RNase H1 (RH1), GFP and Tubulin (loading control) in HCT116-RH2B cells +/- 48 h treatment with doxycycline and 24 h transfection with vectors encoding for GFP, RH1 or RH2A as indicated. **(D)** Slot blot analysis of RNA:DNA hybrid levels in genomic DNA after treatment as in C. S9.6: RNA:DNA hybrids; dsDNA: double-stranded DNA (loading control). **(E)** Quantification of slot blot analysis in HCT116 cells as in D. S9.6 intensities were normalised to dsDNA and to GFP - RH2B. N=3. **(F)** Protein levels of RH2A, RH2B, RH2C, and Tubulin (loading control) in HCT116 after 72 h transfection with non-targeting control siRNA (NTC) or siRNA against RH2B and 50 min treatment with 200 μM hydroxyurea (HU), 10 μM CPT or DMSO. **(G)** Slot blot analysis of RNA:DNA hybrid levels in genomic DNA after treatment as in F. **(H)** Quantification of slot blot analysis in HCT116 cells as in D. S9.6 intensities were normalised to dsDNA and to NTC + DMSO. N=6.

The means and SEM (bars) of independent experiments are shown. Asterisks indicate p-values (ANOVA, ns: not significant, * p < 0.05, ** p < 0.01).

**Figure S5. Impact of RNASEH2B overexpression on gene expression.**

(**A)** RT-qPCR analysis of *CBX5* expression in HCT116-RH2B cells after 48 h RH2B induction. N=3. **(B)** RT-qPCR analysis of *EPCAS* expression in HCT116-RH2B cells after 48 h RH2B induction. N=3. **(C)** Top enriched gene sets that are specifically upregulated upon 48 h dox treatment in inducible HCT116-RH2B cells, but not in parental controls. **(D)** Top enriched gene sets that specifically downregulated upon 48 h dox treatment in inducible HCT116-RH2B cells, but not in parental controls.

The means and SEM (bars) of independent experiments are shown. Asterisks indicate p-values (student’s t-test, ns: not significant).

**Figure S6. Growth characteristics of cells overexpressing RNASEH2B.**

**(A)** Growth curve of BJ-RH2B cells after RH2B induction or no DOX control (con), measured by cell number. N=1. **(B)** Quantification of cell cycle distribution in BJ-RH2B cells after 7 days or 10 days RH2B induction or no DOX control (con), based on measuring DNA content using propidium iodide staining and flow cytometry. N=1. **(C)** Flow cytometry gating strategy for quantification of cell cycle distribution as in B. Cells were stained with propidium iodide. Data from one experiment are shown.

**Figure S7. Impact of RNASEH2B overexpression on colony survival and genomic instability.**

**(A)** Colony survival of HCT116-RH2B cells after continuous treatment with CPT. N= 2. **(B)** Colony survival of HCT116-RH2B cells after continuous treatment with HU. N = 4. **(C)** Percentages of HCT116-RH2B cells with micronuclei +/- RH2B induction +/- 2 mM HU. N=4. **(D)** Percentages of HCT116-RH2B cells with micronuclei +/- RH2B induction +/- 1 μM GEM. N=3. **(E)** Percentages of HCT116-RH2B cells with micronuclei +/- RH2B induction +/- 10 μM CPT. N=3. **(F)** Treatment schematic for micronucleus quantification in BJ-RH2B cells. **(G)** Percentages of BJ-hTERT-RH2B cells with micronuclei after RH2B induction and 24 h treatment with 10 μM CPT, 1 μM GEM, 200 μM HU or DMSO, and release from drug for 24 h. N=3. **(H)** Fold increase in BJ-hTERT-RH2B cells with micronuclei after RH2B induction and 24 h treatment with 10 μM CPT, 1 μM GEM, 200 μM HU or DMSO, and release from drug for 24 h. N=3.

The means and SEM (bars) of independent experiments are shown. Asterisks indicate p-values (ANOVA, ns: not significant, * p < 0.05, ** p < 0.01, *** p < 0.001, **** p < 0.0001).

**Figure S8. Impact of RNASEH2B overexpression on cytoplasmic nucleic acid signalling.**

**(A)** Percentages of BJ-hTERT-RH2B cells with cytoplasmic ssDNA or dsDNA staining +/- 48 h RH2B induction and 24 h treatment with 1 μM CPT, 25 nM GEM, 200 μM HU or DMSO. Circles: dsDNA antibody, squares: ssDNA antibody. N=4 (CPT and GEM), N=3 (HU). **(B)** Fold increase in percentages of HCT116-RH2B cells with cytoplasmic ssDNA or dsDNA staining +/- 48 h RH2B induction and 24 h treatment with 1 μM CPT, 25 nM GEM, 200 μM HU or DMSO. Circles: dsDNA antibody, squares: ssDNA antibody. N=5. **(C)** Percentages of HCT116-RH2B cells with cytoplasmic ssDNA or dsDNA staining +/- 48 h RH2B induction and 24 h treatment with 1 μM CPT, 25 nM GEM, 200 μM HU or DMSO. Circles: dsDNA antibody, squares: ssDNA antibody. N=5. **(D)** RT-qPCR analysis of *IL6* (Interleukin 6) expression in BJ-RH2B cells +/- 72 h RH2B induction and 48 h treatment with 1 μM CPT, 25 nM GEM, 200 μM HU or DMSO. Values were normalised to – RH2B, + DMSO only. N = 3. **(E)** RT-qPCR analysis of *ISG15* expression in BJ-RH2B cells +/- 72 h RH2B induction and 48 h treatment with 1 μM CPT, 25 nM GEM, 200 μM HU or DMSO. Values were normalised to DMSO for each condition. N=3. **(F)** RT-qPCR analysis of *ISG15* expression in BJ-RH2B cells +/- 72 h RH2B induction and 48 h treatment as in E. Values were normalised to – RH2B + DMSO only. N = 3.

The means and SEM (bars) of independent experiments are shown. Asterisks indicate p-values (ANOVA, ns: not significant, * p < 0.05).

**Figure S9. RNASEH2B depletion exacerbates growth arrest and cell death in presence of oncogenic HRAS^G12V^.**

**(A)** Relative proliferation after 8 days transfection with non-targeting control or RH2B siRNA and HRAS^G12V^ induction for the times indicated. Cell counts were normalised to non-targeting control (NTC) without HRAS^G12V^ induction. N = 3.

**(B)** Flow cytometry gating strategy for quantification of sub-G1 phase populations. Cells were stained with propidium iodide. The means and SEM (bars) of independent experiments are shown. Asterisks indicate p-values (ANOVA, ns: not significant, * p < 0.05).

**Supplemental Tables**

| **Figure 4B** |  | **0 days** | **2 days** |  |  |
| --- | --- | --- | --- | --- | --- |
|  | 1 | 257 | 154 |  |  |
|  | 2 | 126 | 122 |  |  |
|  | 3 | 254 | 212 |  |  |
| **Figure 4C** |  | **0 days** | **2 days** |  |  |
|  | 1 | 257 | 154 |  |  |
|  | 2 | 126 | 122 |  |  |
|  | 3 | 254 | 212 |  |  |
|  | 4 | 95 | 171 |  |  |
|  | 5 | 180 | 131 |  |  |
|  | 6 | 77 | 158 |  |  |
| **Figure 4E** |  | **con DMSO** | **RH2B DMSO** | **con CPT** | **RH2B CPT** |
|  | 1 | 62 | 124 | 68 | 116 |
|  | 2 | 95 | 171 | 212 | 191 |
|  | 3 | 180 | 131 | 138 | 216 |
| **Figure 4F** |  | **con DMSO** | **RH2B DMSO** | **con CPT** | **RH2B CPT** |
|  | 1 | 190 | 303 | 202 | 275 |
|  | 2 | 580 | 468 | 737 | 415 |
|  | 3 |  | 635 | 311 | 483 |
|  | 4 | 184 | 415 | 666 | 541 |
| **Figure 4G** |  | **con DMSO** | **RH2B DMSO** | **con CPT** | **RH2B CPT** |
|  | 1 | 215 | 346 | 610 | 449 |
|  | 2 | 268 | 262 | 278 | 701 |
|  | 3 | 352 | 854 | 1663 | 1265 |
| **Figure 4H** |  | **con** | **RH2B** | **con HU** | **RH2B HU** |
|  | 1 | 277 | 536 | 1802 | 1331 |
|  | 2 | 30 | 102 | 349 | 1188 |
|  | 3 | 347 | 295 | 630 | 475 |
| **Figure 4J** |  | **DMSO** | **DRB** | **DMSO CPT** | **DRB CPT** |
|  | 1 | 509 | 662 | 414 | 579 |
|  | 2 | 400 | 275 | 467 | 599 |
|  | 3 | 508 |  | 558 | 431 |
| **Figure 4K** |  | **con HU** | **RH2B HU** | **con TRIP HU** | **RH2B TRIP HU** |
|  | 1 | 253 | 350 | 230 | 295 |
|  | 2 | 205 | 183 | 182 | 235 |
|  | 3 | 259 | 215 | 230 | 203 |
| **Figure 4N** |  | **con HU** | **RH2B HU** |  |  |
|  | 1 | 927 | 829 |  |  |
|  | 2 | 752 | 399 |  |  |
|  | 3 | 447 | 1020 |  |  |
| **Figure 4M, O** |  | **con HU** | **RH2B HU** |  |  |
|  | 1 | 507 | 265 |  |  |
|  | 2 | 189 | 128 |  |  |
|  | 3 | 163 | 224 |  |  |
| **Figure 7D** |  | **NTC con** | **RH2B con** | **NTC HRAS** | **RH2B HRAS** |
|  | 1 | 148 | 77 | 207 | 417 |
|  | 2 | 97 | 188 | 131 | 371 |
|  | 3 | 122 | 198 | 102 | 215 |
| **Figure 7E** |  | **NTC con** | **RH2B con** | **NTC HRAS** | **RH2B HRAS** |
|  | 1 | 148 | 77 | 261 | 222 |
|  | 2 | 97 | 188 | 140 | 177 |
|  | 3 | 122 | 198 | 115 | 224 |

**Table S1: Number of DNA fibres analysed per figure panel and repeat**
